# The genome sequence of ectromelia virus Naval and Cornell isolates from outbreaks in North America

**DOI:** 10.1016/j.virol.2014.06.010

**Published:** 2014-08

**Authors:** Carla Mavian, Alberto López-Bueno, Neil A. Bryant, Kathy Seeger, Michael A. Quail, David Harris, Bart Barrell, Antonio Alcami

**Affiliations:** a Centro de Biología Molecular Severo Ochoa (Consejo Superior de Investigaciones Científicas-Universidad Autónoma de Madrid), Nicolas Cabrera 1, Campus de Cantoblanco, Madrid, Spain; b Department of Medicine, University of Cambridge, United Kingdom; c Wellcome Trust Sanger Institute, Genome Campus, Hinxton, United Kingdom

**Keywords:** Poxvirus, Ectromelia virus, Genome sequence, Virulence

## Abstract

Ectromelia virus (ECTV) is the causative agent of mousepox, a disease of laboratory mouse colonies and an excellent model for human smallpox. We report the genome sequence of two isolates from outbreaks in laboratory mouse colonies in the USA in 1995 and 1999: ECTV-Naval and ECTV-Cornell, respectively. The genome of ECTV-Naval and ECTV-Cornell was sequenced by the 454-Roche technology. The ECTV-Naval genome was also sequenced by the Sanger and Illumina technologies in order to evaluate these technologies for poxvirus genome sequencing. Genomic comparisons revealed that ECTV-Naval and ECTV-Cornell correspond to the same virus isolated from independent outbreaks. Both ECTV-Naval and ECTV-Cornell are extremely virulent in susceptible BALB/c mice, similar to ECTV-Moscow. This is consistent with the ECTV-Naval genome sharing 98.2% DNA sequence identity with that of ECTV-Moscow, and indicates that the genetic differences with ECTV-Moscow do not affect the virulence of ECTV-Naval in the mousepox model of footpad infection.

## Highlights

• We describe the genome sequence of two highly virulent ectromelia virus isolates.
• The outbreak of ectromelia virus in USA was caused by Chinese viral isolates.
• We describe a clade of ectromelia virus isolates from China.
• We compare three different sequencing technologies to sequence large DNA viruses.

## Introduction

Mousepox is an acute exanthematous disease of mice caused by the orthopoxvirus (OPV) ectromelia virus (ECTV) that in the past century affected laboratory mouse colonies worldwide (Esteban and Buller, 2005, Fenner, 1981, Marchal, 1930). ECTV, like variola virus (VARV), the causative agent of smallpox, has a narrow host range and causes a severe disease with skin lesions in the later stages of the infection and a high mortality rate (Esteban and Buller, 2005). Mousepox was first described in 1930 in Hampstead (United Kingdom) as an infectious disease of mouse associated with high mortality and amputations in mice recovered from infection (Marchal, 1930). Following the identification of ECTV-Hampstead, ECTV has been isolated in temporally and geographically different laboratory mousepox outbreaks. ECTV-Moscow is a highly virulent strain isolated in Moscow in 1947 and extensively used in pathogenesis studies, and ECTV-Ishibashi, isolated in Japan in 1966, was used in the initial ECTV studies (Allen et al., 1981, Andrewes and Elford, 1947, Ichihashi and Matsumoto, 1966). Several important mousepox outbreaks occurred in the USA between the seventies and the eighties. As a consequence, tens of thousands of laboratory mice were sacrificed and millions of dollars were lost in biomedical research (Lipman et al., 1999). ECTV-NIH79 was a virulent strain isolated in 1979 at the National Institutes of Health (NIH) in Bethesda, Maryland (USA) (Allen et al., 1981). In the same period, various outbreaks were reported in biomedical research institutions in Saint Louis, Chicago, Minneapolis and Salt Lake City (Dixon, 1981, La Regina and Doyle, 1981, Wallace, 1981). Because the clinical signs of mousepox are not evident in resistant mouse strains, ECTV infections could have been present in colonies for long periods before their identification in animal house facilities in USA. The destruction of entire mouse colonies was not always satisfactory to control mousepox spread (Wallace, 1981). In order to fight this continuous threat, working with live ECTV was prohibited in the USA (Fenner et al., 1989). In 1995, the virulent strain ECTV-Naval caused an outbreak in the laboratory mouse colony of the US Naval Medical Research Institute in Bethesda, Maryland (USA) (Dick et al., 1996). The outbreak caused devastating effects and ended with the sacrifice of thousands of mice, affecting more severely the BALB/c mice (Dick et al., 1996). The last outbreak of mousepox was reported in 1999 at the Weill Medical College of Cornell University in New York (USA), and was successfully contained and eradicated from the animal house facility (Lipman et al., 1999, Lipman et al., 2000). Contrary to initial speculations about the existence of a natural reservoir for this virus within the USA, it was found that the Cornell University outbreak originated from an injection of mice with a contaminated commercial mouse serum imported in 1995 from China, and the same origin was speculated for the outbreak in Bethesda that occurred four years earlier (Lipman et al., 1999, Lipman et al., 2000). ECTV outbreaks have no longer been reported (Huggins et al., 2009), probably due to improved housing conditions of laboratory mouse colonies and routine testing of the health status of mouse colonies. Nearly a century after the first ECTV description, it is intriguing that this virus has never been isolated from a mouse or rodent in nature, and its natural reservoir remains unknown (Fenner et al., 1989). The only exception was a report describing a putative ECTV infection in field mice in Germany in 1962. This disease could be transmitted by footpad inoculation to laboratory white mice and produced typical symptoms of ECTV infection, including skin lesions, foot inflammation and amputation (Groppel, 1962). However, the virus was not isolated and the presence of ECTV was not confirmed by molecular techniques.

The growing fear of the possible use of VARV as a bioterrorist weapon causing an epidemic in the human population that is no longer vaccinated against the disease prompted the need for the development of safer smallpox vaccines (Smith and McFadden, 2002). Moreover, OPV zoonoses have increased in recent years; monkeypox virus (MPXV) may occupy the niche vacated by VARV in Africa and cowpox virus (CPXV) is transmitted to humans by domestic animals and occasionally wild rodents in Europe (Favier et al., 2011, Giulio and Eckburg, 2004, Wolfs et al., 2002). The major limitation for the improvement of the current repertoire of poxvirus vaccines is the lack of accessible human OPV infections for clinical efficacy trials. The genetic similarity of ECTV to VARV, CPXV and MPXV, together with the convenience of a laboratory mouse model, underscores the utility of mousepox as a model for the study of OPV infections (Buller, 2004).

The recent development of next generation sequencing technologies has had a pronounced impact on virology providing a fundamental tool for sequencing large viral genomes (Afonso et al., 2012, Lin et al., 2013, Mavian et al., 2014, Mavian et al., 2012a, Mavian et al., 2012b, Qin et al., 2013) for metagenomic studies of viral communities (Lopez-Bueno et al., 2009, Reyes et al., 2010) and also for the identification of new viruses and pathogens (Radford et al., 2012). In spite of the relevance of the mousepox model, only two ECTV genomes have been sequenced: ECTV-Moscow and Erythromelalgia-related poxvirus (ERPV), an ECTV strain isolated in China from throat specimen of individuals affected by erythromelalgia, a vascular disease of extremities and characterized by hyperthermia and vasodilatation (Chen et al., 2003, Mendez-Rios et al., 2012, Zheng et al., 1992, Zheng et al., 1988). Here we report the sequence of the ECTV-Naval and ECTV-Cornell genomes determined by three different sequencing technologies (Sanger, 454-Roche and Illumina) and define the virulence of these isolates in susceptible mice.

## Results

### Plaque purification of ECTV-Naval.Cam

The plaque-purified ECTV-Naval.Cam stock was obtained after three rounds of plaque purification of a single plaque derived from the spleen of a BALB/c mouse infected with the original stock of the Naval Medical Research Institute outbreak (Bethesda, Maryland, USA) (Dick et al., 1996). The virulence in susceptible BALB/c mice of ECTV-Naval.Cam is similar to that of the original uncloned stock, indicating that the plaque-purified virus has not been attenuated and is representative of the parental virus isolate (data not shown). ECTV-Naval.Cam is referred in the text as ECTV-Naval.

### Sequencing of the ECTV-Naval genome

The genome of ECTV-Naval has been sequenced by three different sequencing technologies: Sanger, 454-Roche and Illumina. Sequences obtained by Sanger (5810 reads with an average length of 536 bp) were assembled into five contigs accounting for 202 Kbp. Further 42 reads were obtained to close gaps and to raise the quality of low coverage regions. Finally, a contiguous linear sequence of 207,620 bp with 13× coverage was attained (Table 1). The leftmost nt was an arbitrarily designated base one. The ITR of the genome contains the OPV specific structures: the hairpin loop, the concatemer resolution motif, two sets of direct repeats (DRI and DRII) and the non-repetitive I (NRI) and NRII sequences flanking DRI. The DRI of the ECTV-Naval genome, as for ECTV-Moscow, is formed by a unique sequence of 68 bp, in comparison with the 69 bp sequence repeated 2.3× of ERPV (Chen et al., 2003, Mendez-Rios et al., 2012). DRII contains an 85 bp sequence repeated 19.4×, instead of 10.4× found in the ECTV-Moscow and ERPV genomes (data not shown) (Chen et al., 2003, Mendez-Rios et al., 2012). A third set of direct repeats, located in the central region of the genome (DRIII), contained a 24 bp (“TCTATATCCTGTACTATACCATTA”) sequence repeated 25×. Although the hairpin loop structure was not experimentally isolated and sequenced, the comparison by clustalw2 alignment with the sequence of the termini of the ECTV-Moscow and ERPV genomes suggested that the genome was represented completely in the sequence, including the hairpin loop structures.

**Table 1:** DNA sequencing of the ECTV-Naval and the ECTV-Cornell genomes.

	ECTV-Naval			ECTV-Cornell
	Sanger	454-Roche	Illumina	454-Roche
Total reads	5810	92,155	5,497,834	67,965
Mapped reads (%)^a^	nd	45,195	4,835,589	58,558
		(49.0%)	(87.9%)	(86.1%)
Average reads size (bp)	536	360	76	384
Genome coverage (x)	13	83	1770	115
Genome size (bp)	207,620	204,099	207,620	204,499
Differences with Sanger genome b	–	8	0	nd

nd, not determined.

a Number of reads mapped to the reference viral genome .

b Number of nucleotide differences as compared to the Sanger genome.

**Fig. 1:**
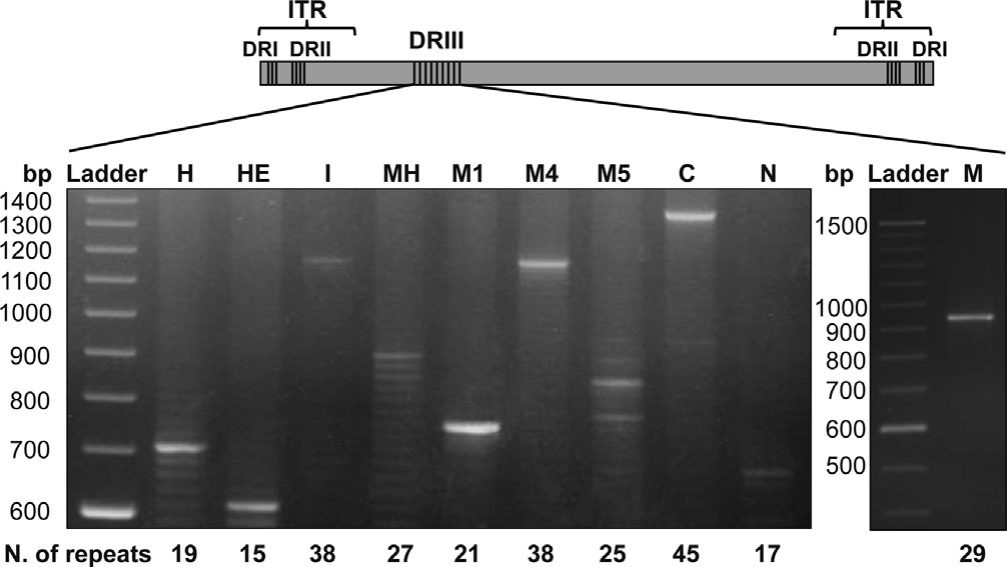
Variability of the DRIII region among ECTV strains. (A) The locations of the DRI, DRII and DRIII regions in the schematic representation of the ECTV genome are shown. The DRIII region was PCR-amplified from genomic DNA and analyzed by 2% agarose gel electrophoresis. Molecular size markers (Ladder) are shown. (B) The estimated number of 24 bp repeats for each ECTV strain is shown. H, ECTV-Hampstead; HE, ECTV-Hampstead Egg; I, ECTV-Ishibashi; MH, ECTV-Mill Hill; M1, ECTV-MP1; M4, ECTV-MP4; M5, ECTV-MP5; C, ECTV-Cornell; N, ECTV-Naval; M, ECTV-Moscow.

The 454-Roche sequencing output consisted of 92,155 reads with an average length of 360 bp (Table 1). The 454-Roche reads were assembled into two contigs of approximately 35 Kbp and 163 Kbp, separated by the DRIII region. Sanger sequencing of a PCR product including the DRIII region also failed to determine the exact number of repeats. We estimated by gel electrophoresis of the PCR product the presence of 17 repeats of 24 bp in the DRIII region (Fig. 1). The genome sequence obtained by 454-Roche has a size of 204,205 bp, including a DRII region of 85 bp sequence repeated only 2.3× (Table 1). The coverage of the genome was 83×, almost seven times higher than the coverage obtained by Sanger. This 454-Roche genome showed eight differences when compare to the genome obtained by Sanger, mostly due to A/T homopolymeric regions.

The genome sequence determined by Illumina was 207,620 bp long with a coverage of 1770×, which was 21 times and 147 times higher than those obtained by 454-Roche and Sanger sequencing, respectively (Table 1). The genome determined by Illumina was identical in sequence to that obtained by Sanger.

In conclusion, the ECTV-Naval genome reported here corresponds to the genome sequence obtained by Sanger and Illumina, but considering the 17 repeated sequences in the DRIII region estimated by gel electrophoresis (Fig. 1). With a total size of 207,428 bp, this genome contains an ITR of 7377 bp and shows an A/T typical OPV composition of 66.87%.

### Variability in the number of repeats in the DRIII region among ECTV isolates

Since the DRIII region appeared to be highly variable, we compared the length variation of this region in a collection of ECTV isolates available in our laboratory (Smith and Alcami, 2000): (i) ECTV-Moscow, plaque-purified from the virus isolated in an outbreak in Moscow in 1947 (Andrewes and Elford, 1947); (ii) ECTV-Hampstead, the first ECTV isolated in 1930 from London (Marchal, 1930); (iii) ECTV Hampstead-Egg and ECTV-Mill Hill, derived from the ECTV Hampstead isolate by serial passage in eggs (Smith and Alcami, 2000); (iv) ECTV-Ishibashi, a plaque purified virus derived from an outbreak in Japan in 1966 (Ichihashi and Matsumoto, 1966); (v) two isolates ECTV-MP1 (München, 1976) and ECTV-MP4 (Nürnberg, 1976) from German outbreaks (Mahnel, 1983, Osterrieder et al., 1994); (vi) ECTV-MP5 from an outbreak in Wien (Austria) in 1994 (Osterrieder et al., 1994); and (vii) ECTV-Cornell isolated in the most recent outbreak in the USA (Lipman et al., 2000). A high variability in the number of repeats was detected among all the ECTV strains (Fig. 1) ranging from 15 repeats for ECTV Hampstead-Egg to 45 repeats estimated for ECTV-Cornell. Interestingly, different number of repeats were determined by PCR amplification followed by agarose gel analysis and by Sanger sequencing for ECTV-Naval (17 vs. 25 repeats, respectively) and for ECTV-Moscow (29 vs. 30 repeats, respectively) (Chen et al., 2003). An alternative method to estimate the number of repeats in DRIII was to consider the number of Illumina sequencing reads aligned to this region compared to the number of sequencing reads in flanking non-repetitive regions of the genome. We estimated in this way the presence of 15 repeats in the DRIII region of the ECTV Naval genome, which was very close to the 17 repeats estimated by PCR amplification and agarose gel.

### Annotation of the ECTV-Naval genome

**Fig. 2:**
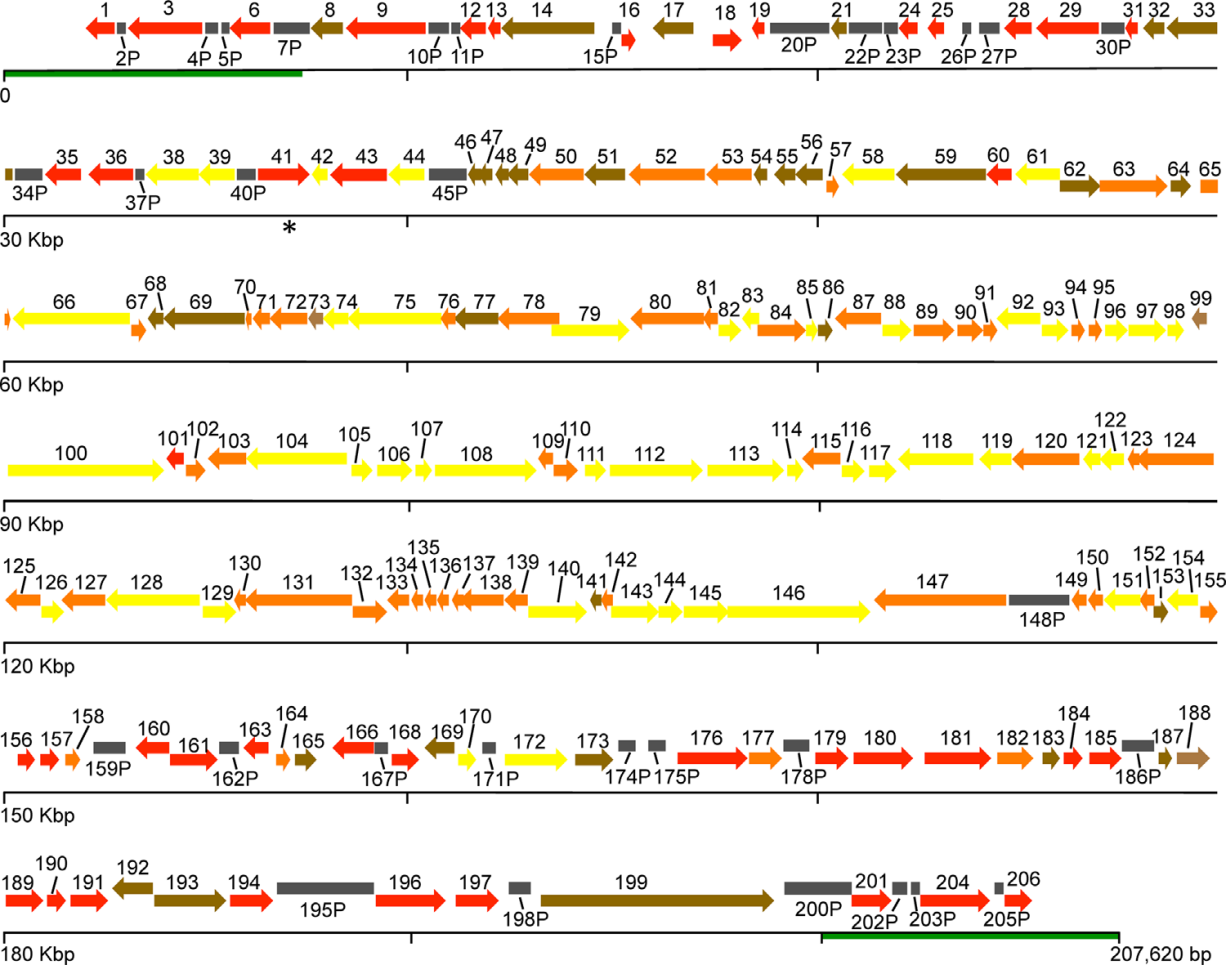
Schematic organization of the ECTV-Naval genome. Predicted putative genes are represented as arrows indicating the approximate size and direction of transcription. The color of the arrows indicate the predicted function: red, immunomodulatory and host range; yellow, metabolism; orange, structural proteins and morphogenesis; brown, unknown function. Pseudogenes are indicated as dark gray rectangles. ITRs are marked as a green line.

Non-overlapping ORFs, with a minimum of 120 nt, encoding for proteins with similarity to a poxvirus ortholog of known function or conserved domains, were annotated as genes. Genes that were fragmented due to the presence of mutations, deletions or insertions that interrupted translation of the protein were designated as pseudogenes and named with a ‘P’. Genes as well as pseudogenes were given a number according to their position in the genome (Fig. 2, Table S1). A total of 206 putative genes were annotated in the ECTV-Naval genome: 174 genes and 32 pseudogenes. All genes shared more than 90% identity with the orthologs from the vaccinia virus strain Copenhagen (VACV-Cop), CPXV-BR or VARV-BSH reference genomes (Table S1). The genome of ECTV-Naval showed the typical poxviral genomic organization with essential genes in the central region of the genome and genes involved in virus-host interaction in the terminal regions of the genome.

The conserved region of the genome of ECTV-Naval includes essential genes that are involved in DNA replication and RNA transcription (47 genes) or morphogenesis (53 genes) (Fig. 2, Table S1). It has been reported that 90 genes in the central region of VACV-Cop, from *F9L* to *A34R*, define the minimal gene content conserved in all the chordopoxvirus genera (Lefkowitz et al., 2006). These genes in the ECTV-Naval genome, from *EVN049* to *EVN156*, are conserved in VACV-Cop and VARV-BSH, with a few exceptions. For example, *EVN064* and *EVN136* are absent in VARV-BSH; *EVN147*, that encodes for the A-type inclusion body protein (ATIp), is present in CPXV-BR but fragmented in VACV-Cop and VARV-BSH; and *EVN148P*, a pseudogene of the *P4c* gene, is fragmented in CPXV-BR whereas it is a full-length gene in VACV-Cop and VARV-BSH (Table S1).

Genes that modulate the host immune system or determine the host range (42 genes) are located in the variable terminal regions of the genome (Fig. 2, Table S1). These regions include genes encoding the viral growth factor, semaphorin homolog, ankyrin-like and kelch-like proteins involved in determining host range, inhibition of the ubiquitin proteasome system and apoptosis (Seet et al., 2003, Shchelkunov, 2012), and several secreted proteins which modulate inflammatory responses by sequestering cytokines such as receptor/binding proteins for chemokines, interleukin (IL)-1β, interferon (IFN)-α/β, IFN-γ and tumor necrosis factor (TNF) (Alcami et al., 1998, Graham et al., 1997, Loparev et al., 1998, Sakala et al., 2007, Smith et al., 1997, Smith and Alcami, 2000, Smith and Alcami, 2002, Xiang and Moss, 1999, Xu et al., 2008) (Table S1). A total of 33 genes have no function ascribed (Fig. 2, Table S1).

Seventeen pseudogenes are present in the right terminal region of the genome, 14 in the left terminal region and only one (*EVN148P*) in the central region of the genome (Fig. 2, Table S1). Three genes and three pseudogenes are found in the ITR, which are consequently diploid. The duplicated genes encode for a chemokine binding protein (35 kDa), a TNF receptor/chemokine binding protein (Cytokine response modifier D, CrmD) and an ankyrin-like protein that blocks NF-*k*B activation (Alcami et al., 1998, Graham et al., 1997, Loparev et al., 1998, Rubio et al., 2013). Interestingly, whereas CPXV encodes four TNF receptors (CrmB, CrmC, CrmD and CrmE), ECTV conserves only one functional protein, CrmD encoded by the *EVN006* gene, and the genes encoding CrmB, CrmE and CrmC are mutated, corresponding to pseudogenes *EVN002P*, *EVN005P* and *EVN175P*, respectively (Alejo et al., 2011) (Fig. 2, Table S1). The pseudogene *EVN007P* has not been fully duplicated laying in the intersection between the ITRs and the unique sequence.

Comparison with the ECTV-Moscow genome annotation showed several differences (Tables S1 and S2) (Chen et al., 2003). The ECTV-Naval *EVN071* gene is a new ORF that was not annotated in the ECTV-Moscow genome. The ortholog of this novel gene in VACV-Cop, *O3L*, has been recently described to encode a small protein of 35 amino acids implicated in the formation of the entry/fusion complex (Satheshkumar and Moss, 2009). The *EVN010P* pseudogene is formed by two fragments of the same gene that in ECTV-Moscow has been annotated as separate and independent gene (Chen et al., 2003). These fragments show sequence similarity to the C-type lectin encoded by CPXV-Germany 91-3 (ABD97357) (data not shown). The truncated version of this protein in CPXV-BR, CPXV012, is responsible for down-regulation of major histocompatibility complex class I molecules (Alzhanova et al., 2009, Byun et al., 2007). Despite sharing a 67% of amino acid sequence similarity with the first 46 amino acids of the CPXV012 protein, neither the truncated protein from ECTV-Moscow, nor the smaller protein from ECTV-Naval seem to be functional (Alzhanova et al., 2009, Byun et al., 2007). The ortholog of *EVM144*, encoding a superoxide dismutase in VACV-Cop *A45R* (Almazan et al., 2001, Cao et al., 2002) has lost 18 amino acids from its C-terminus in ECTV-Naval, and therefore has been annotated as pseudogene *EVN167P*. This truncation is due to the deletion of an adenine in a homopolymeric region that has been confirmed by the Sanger and Illumina sequencing. The ECTV-Naval gene *EVN019* codes for an IL-18 binding protein of 126 amino acids, whereas its ECTV-Moscow ortholog *EVM013* is 12 amino acids longer. The IL-18 binding protein encoded by both ECTV-Naval and ECTV-Moscow have been shown to be active (Esteban and Buller, 2004, Smith and Alcami, 2000). The ECTV-Naval *EVN038* gene, that codes for a DNAse with a size of 424 amino acids, is truncated (372 amino acids) in the ECTV-Moscow genome and it was annotated as a pseudogene (Region N) (Chen et al., 2003). Differences between ECTV-Naval and ECTV-Moscow also include some amino acid mutations in a number of proteins (Table S2). In summary, the ECTV-Naval genome has 98.2% sequence identity with the ECTV-Moscow genome, and the predicted functional differences are limited to a shorter IL-18 binding protein (*EVN019*), a truncated superoxide dismutase (*EVN167P*) and a longer DNAse (*EVN038*) in ECTV-Naval, in addition to amino acid mutations in several proteins (Tables S1 and S2).

Unlike the recent ERPV annotation, but similar to the ECTV-Moscow annotation, we have annotated *EVN026P* and *EVN174P* as pseudogenes. *EVN026P* encodes only 106 amino acids of the 204 amino acids of its VACV-Cop ortholog *C5L*, having lost the kelch/BTB domain. This pseudogene is identical to that of ECTV-Moscow, annotated as ‘Region I’ and to the annotated gene *ERPV017* of ERPV. *EVN174P* is an ortholog of VACV-Cop *A52R*, an inhibitor of intracellular signaling mediated by IL-1, IL-18 and Toll-like receptors, and codes for a 33% smaller protein (Baxby, 1982, Bowie et al., 2000, Harte et al., 2003) (Table S1).

### The ECTV-Cornell genome: sequencing, annotation and comparison with the ECTV-Naval genome

The ECTV-Cornell isolate, obtained from the Weill Medical College of Cornell University outbreak (Lipman et al., 2000), was not plaque-purified. The ECTV-Cornell genome was sequenced by the 454-Roche technology and, like the ECTV-Naval genome, was assembled into two contigs separated by the DRIII region consisting of 45 repeats of a 24 bp sequence, as estimated by gel electrophoresis (Fig. 1).

The ECTV-Cornell genome has a size of 204,499 bp with a coverage of 115×, including two copies of the 85 bp sequence of the DRII region (Table 1). The initial annotation of the ECTV-Cornell revealed that three genes (*EVC019*, *EVC077* and *EVC133*) were interrupted due to incorrect estimation of the length of A/T homopolymers. PCR amplification of homopolymeric regions was carried out to determine the exact number of A/Ts and confirmed that full-length genes are also present in ECTV-Cornell (data not shown).

The ECTV-Naval and ECTV-Cornell genomes are almost identical to each other with a 99.9% identity, differing only in four positions of the genome (Table 2); the number of 24 nt repeats determining a different number of “DIDNGIVQ” in the *EVN041*/*EVC041* genes, two single nt synonymous changes affecting genes *EVN160*/*EVC160* and *EVN165/EVC165*, and a different size in the homopolymer of the position 155,375 of the ECTV-Naval genome affecting the *EVN162P*/*EVC162P* pseudogenes. The ECTV-Naval and ECTV-Cornell genomes are very similar to the genome of ERPV, differing only in 12 positions, nine of which are affecting putative genes (Table 2).

**Table 2:** Differences among the genomes of ECTV-Naval, ECTV-Cornell and ERPV.

**Position in ECTV-Naval**	**ORF**	**ECTV-Naval**	**ECTV-Cornell**	**ERPV**	**Amino acid differences**
36,910	EVN041	17×24 pb	45×24 pb	7×24 pb	Variable number of “DIDNGIVQ”
55,612–55,613	EVN062	–	–	AT	10 aa N-terminal deletion
130,142	EVN081	T	T	C	V66A
127,552	EVN131	A	A	G	R236G
125,616	EVN131	T	T	C	V881A
152,911	EVN160	T	C	C	–
155,375	EVN162P	11×C	9×C	7×C	–
158,361	EVN165	G	T	G	–
159,104	EVN166P	5×A	5×A	6×A	–
159,383	EVN167	T	T	C	S67P
167,577	EVN176	A	A	G	N358D
168,661	EVN177	T	T	G	Y139D
189,833	EVN196	A	A	G	M241V

### Virulence of ECTV-Naval and ECTV-Cornell

**Fig. 3:**
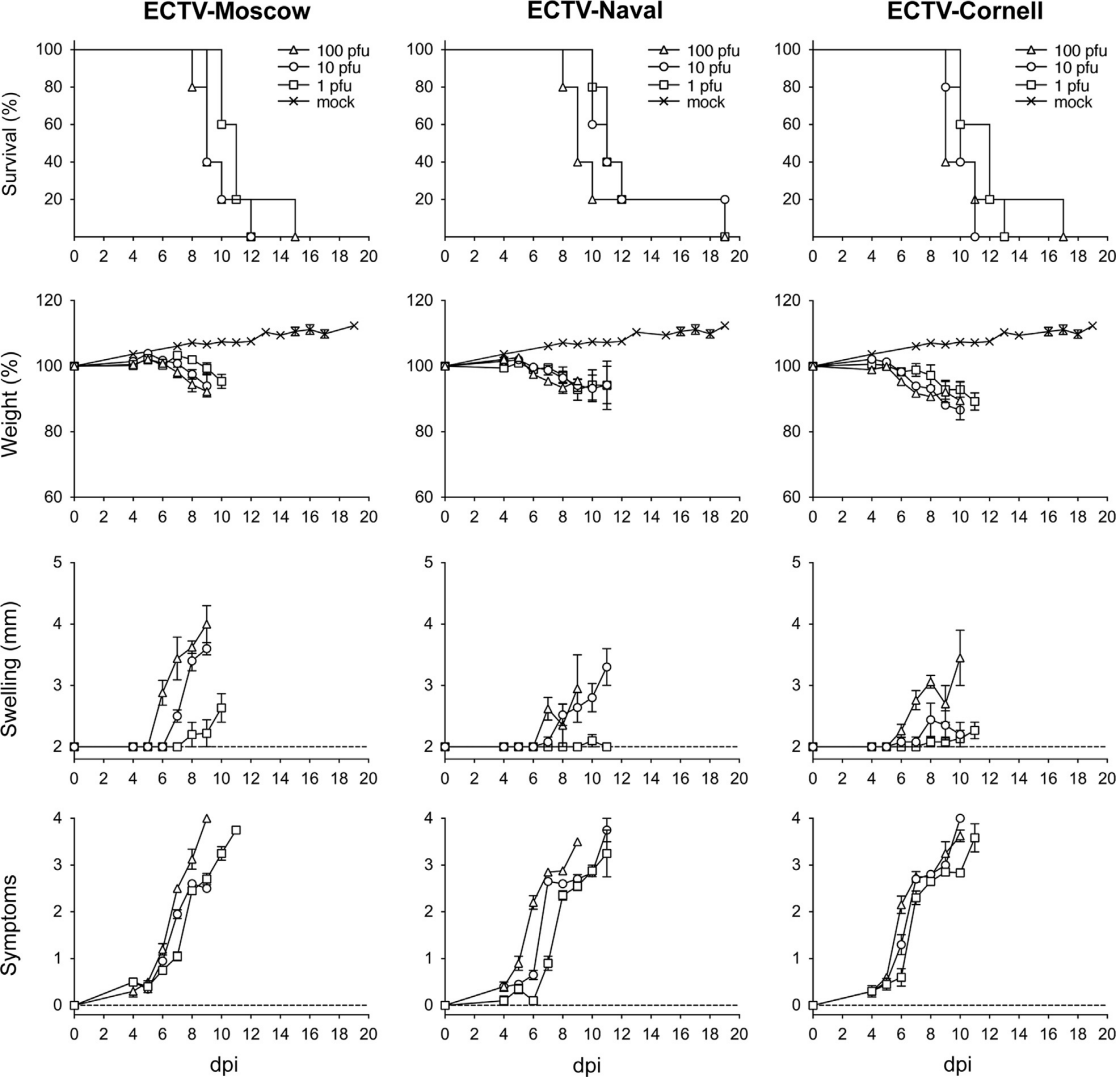
Virulence of the ECTV-Moscow, ECTV-Naval and ECTV-Cornell isolates in BALB/c mice. Groups of five female 5–6 week old mice were subcutaneously infected in the footpad with different doses of ECTV-Moscow (A), ECTV-Naval (B) or ECTV-Cornell (C) (1, 10 and 10^2^ pfu per mouse). Mortality (%), weight (%), swelling of the foot (mm) and signs of illness are represented.

The virulence of ECTV-Naval and ECTV-Cornell in susceptible BALB/c mice was compared to that of ECTV-Moscow. Severe mousepox disease and death was caused by ECTV-Moscow, ECTV-Naval and ECTV-Cornell at 1 pfu per mouse indicating an LD_50_<1 pfu (Fig. 3). The time of death (12–19 dpi) and weight loss (average of 7.2% at 9 dpi) were indistinguishable in mice infected with these viruses. All mice suffered severe signs of illness (hunched posture, conjunctivitis and reduced mobility) but limited foot swelling was observed. With higher doses of 10 and 10^2^ pfu per mouse, mortality and disease progression were similar but foot swelling started 1–2 days earlier in mice infected with ECTV-Moscow. Footpad infection of susceptible DBA/2 mice with 0.1, 1, 10 and 10^2^ pfu of ECTV-Naval or ECTV-Moscow showed a LD_50_<1 pfu for both viruses (data not shown).

## Discussion

ECTV is the causal agent of mousepox, an OPV infection of mice that affected animal facilities in Europe, Asia and USA during the past century. ECTV-Naval and ECTV-Cornell are isolates that caused two separate outbreaks in laboratory mouse colonies in USA. We report the genomic sequence of ECTV-Naval, sequenced with three different technologies (Sanger, 454-Roche and Illumina), and ECTV-Cornell, sequenced with 454-Roche.

The complete genome sequence of ECTV-Naval obtained with the Sanger method or Illumina sequencing were identical, whereas the 454-Roche technology failed to determine the exact number of nt in homopolymers of more than six consecutive A/T bases. Several of these errors in homopolymers caused the introduction of early stop codons in some ORFs. The need to correct these errors by further steps of PCR amplifications and Sanger sequencing limits the use of the 454-Roche technology for *de novo* sequencing or re-sequencing of OPV genomes or any other genome with a high A/T content. We concluded that Illumina appears to be a more solid technology, in terms of quality and cost-benefit when sequencing poxvirus genomes with a close reference genome available. It has been proposed that the 454-Roche technology might be a better choice for *de novo* assembly of poxviral genomes without a related reference genome, due to the longer sequences obtained. However, the recent increase in read length attained with the Miseq platform (2×300 bp), together with its higher coverage, will make this technology most suitable for *de novo* assembly of large viral genomes.

Nevertheless, we found that Sanger, 454-Roche and Illumina present a common limitation. These three technologies are not able to determine the exact number of repeated sequences that cover a portion of the genome larger than the average size of the output reads. To solve this problem we performed a gel electrophoresis estimation of the length of the PCR products and it revealed a high variability in the number of repeats among all the ECTV strains, whereas only a few copies are present in VACV and CPXV (Yu et al., 2011). It is unlikely that these repeats have originated artificially during the Phi29 polymerase amplification step in library production because they were found in the genomes of ECTV sequenced by Sanger: ECTV-Naval (this publication) and ECTV-Moscow (Chen et al., 2003). This variability is likely due to a DNA polymerase slippage event occurring during viral DNA replication, normally described for short tandem repeats of three bases but also possible for longer sequences (Streisinger et al., 1966). This viral DNA polymerase slippage could explain the high variability in the number of repeats found in different ECTV isolates or virus stocks. Indeed, the ECTV-Naval and ECTV-Moscow DNA template used for the Sanger sequencing of the genomes (25 and 30 repeats, respectively) and those used for the PCR gel estimation (17 and 29 repeats, respectively) derived from distinct viral amplifications. A mixture of genomes with different number of repeats was even observed within the same virus stock in our gel analysis of PCR products. The repeats are located within the *EVN041* gene, the ortholog of VACV-Cop *F1L*, and encode numerous reiterations of eight amino acids (“DIDNGIVQ”). Despite this variation in protein length the crystal structure of VACV-Cop F1 indicates that the structural determinants essential for the inhibition of caspase 9 are preserved, and that even if 45 repeats of “DIDNGIVQ” are present, as for ECTV-Cornell, the protein may be active (Yu et al., 2011).

In order to ensure that genes were correctly annotated, we have taken into account recently published data describing novel gene functions, such as the VACV-Cop O3 protein, a component of the entry/fusion complex (Satheshkumar and Moss, 2009), or recently defined structural domains, such as the VACV-Cop F1 protein (Yu et al., 2011). ORFs with mutations that cause the loss of an initiator methionine or the introduction of stop codons and frameshifts leading to the fragmentation of genes otherwise active in other poxviruses were annotated as pseudogenes. These fragmented genes are often found in viral genomes, probably reflecting that the mutations were introduced recently during evolution. This common phenomenon in OPV genomes leads to the identification of small ORFs that correspond to fragments of the active gene or new ORFs derived from translation of an alternative reading frame, even in the reverse orientation to the original gene. The latter is very often misleading because it may be interpreted as the presence of a new gene in a particular virus whereas the reality is that a genuine gene located in that position is fragmented. Pseudogenes highlight the absence of an active gene, information that may be relevant to evaluate the contribution of such gene to viral pathogenesis. Pseudogenes were only annotated when there was clear evidence for the existence of the active ortholog gene, either from comparative analysis of other poxvirus genomes and/or from functional data available in the published literature. When this was not clear or the genes were truncated and could still retain activity, they were annotated as genes. We have named pseudogenes with the corresponding order gene number and added the letter ‘P’ to distinguish them from genes and to simplify a possible change of pseudogene to gene, if its functionality is demonstrated in the future. We believe that this annotation will provide a better interpretation of the coding capacity of poxvirus genomes.

The ECTV-Naval and ECTV-Cornell genomes are almost identical, differing in four silent mutations and in the number of the 24 nt direct repeats. The number of repeats is unlikely to contribute to virulence since they can vary even between stocks of the same ECTV. Moreover, the in vivo experiments showed an LD_50_<1 pfu per mouse for both ECTV-Naval and ECTV-Cornell in BALB/c mice. In light of these results, it is conceivable that ECTV-Naval and ECTV-Cornell are the same virus. Further evidence supporting this hypothesis is that the outbreak at Cornell University was caused by the injection of mice with a contaminated commercial serum imported in 1995 from China, the same year the outbreak occurred in the Naval Medical Research Institute, associated also with the use of a contaminated mouse serum (Dick et al., 1996, Lipman et al., 1999). This suggests that both outbreaks were caused by the same ECTV present in the mouse serum batch imported to the USA at that time. ECTV-Naval shares a 99.8% nucleotide identity with ERPV (Mendez-Rios et al., 2012). The similarity of ERPV to ECTV-Naval, together with the origin of the mouse imported serum, indicate that these viruses originated in China and support the presence of an ECTV reservoir in that country. Several records describing ERPV mention the existence of a previous Chinese ECTV from the early 1960s and at least six isolates of ERPV have been described (Zheng et al., 1992, Zheng et al., 1988).

## Materials and methods

### Cells and viruses

Monkey kidney BS-C-1 cells (ATCC: CCL-26) were grown in Dulbecco׳s modified Eagle׳s medium supplemented with 10% heat-inactivated fetal bovine serum (Gibco BRL). Plaque-purified ECTV-Moscow-3-P2 (ECTV-Moscow) (Chen et al., 2003), derived from the original stock of F. Fenner (Andrewes and Elford, 1947), and ECTV-Naval, the original stock of the Naval Medical Research Institute outbreak (Bethesda, Maryland, USA) (Dick et al., 1996), were provided by R. M. L. Buller (School of Medicine, Saint Louis University). Plaque-purified ECTV-Naval.Cam was derived from ECTV-Naval as follows. A BALB/c mouse was infected with ECTV-Naval and 7 days after infection the spleen was homogenized and several dilutions plated on BS-C-1 cell monolayers. A virus plaque was clonally isolated through three rounds of plaque purification on BS-C-1 cells obtaining ECTV-Naval.Cam. ECTV-Naval.Cam is referred in the text as ECTV-Naval. ECTV-Cornell isolated from the Weill Medical College of Cornell University outbreak (Lipman et al., 2000) was provided by H. Meyer (Institute of Microbiology, Federal Armed Forces Medical Academy, Munich, Germany) and N. Lipmann (Weill Medical College of Cornell University, New York, N.Y.). The source of the ECTV isolates Hampstead, Hamstead-Egg, Mill-Hill, Ishibashi, MP1, MP4 and MP5 are described elsewhere (Smith and Alcami, 2000).

Viral stocks for animal experiments and isolation of DNA were obtained from semi-purified viral particles. Briefly, BS-C-1 cells were infected at a multiplicity of infection of 0.01 plaque forming units (pfu) per cell and homogenized by serial syringe passages. Nuclei and cell debris were discarded by centrifugation at 900*g* for 10 min at 4 °C, and viral particles were isolated by ultracentrifugation at 20,000*g* for 1 h at 4 °C through a 36% sucrose cushion and resuspended in 10 mM Tris pH 9.

### Isolation of viral DNA

Free DNA from semi-purified viral stocks was digested with DNAse I (500  Units/ml) (Roche), Nuclease S7 Micrococcal (500 Units/ml) (Roche) and RNAse A (20 μg/ml) (Roche). After proteinase K (200 μg/ml) (Invitrogen) and 0.5% sodium dodecyl sulfate treatment, viral DNA was extracted with phenol/chloroform and precipitated with 0.1 volume of 3 M sodium acetate pH 5.3 and 2.5 volumes of ethanol in the presence of 10 μg/ml of oyster glycogen (Roche) as carrier. In the case of 454-Roche and Illumina sequencing, viral DNA was subsequently purified by gel electrophoresis with 1% low melting agarose (MicroSieve Clone LM agarose, Flowgen Bioscience) and extracted with the QIAquick Gel Extraction Kit (Qiagen), and purified viral DNA was amplified by multiple displacement amplification using Phi29 DNA Polymerase as described by the manufacturer (Illustra GenomiPhi V2 DNA Amplification Kit; GE Healthcare). Phi29-amplified viral DNA was precipitated with ethanol in order to minimize the primer contamination.

### Genome sequencing and assembly

For Sanger sequencing, purified viral genome was fragmented by shotgun and fractions ranging from 1.4 to 2 Kbp were cloned into pUC18 vector. Random sub-clones were sequenced using dye-terminator chemistry on ABI 377 automated sequencers at the Wellcome Trust Sanger Institute (Hinxton, United Kingdom). The entire sequence was read on both strands and the genome was assembled with PHRAP/GAP4 (http://www.phrap.org/) (Bonfield et al., 1995). For 454-Roche sequencing, 2 μg of the Phi29-amplified viral DNA was used for the construction of a library using 454-Roche GS-FLX Titanium system (454 Life Sciences, Roche). Sequencing reads obtained with a FLX Genome Sequencer in the Parque Científico de Madrid were assembled in three steps with Newbler 2.5.3 (Roche). First, using the GS Reference *Mapper* the reads were aligned to the ECTV-Naval genome obtained by Sanger as reference under the parameters: 90% minimum overlap and 95% identity. Second, a *de novo* assembly was carried out with GS De Novo *Assembler* under more stringent parameters (90% bases overlap and 97% overlap identity) in order to determine the inverted terminal repeat (ITR) regions. Finally, a second mapping was carried out at 97% of identity in at least 90% of the sequence length. For Illumina sequencing, 5 μg of the Phi29-amplified viral DNA were used for the construction of a TruSeq library. Reads obtained with a Genome Analyzer IIx in the Parque Científico de Madrid were mapped using the trial version of CLC-Genomics Workbench 5.5.1 (CLC bio) to the Sanger ECTV-Naval genome as reference under the following parameters: 90% length overlap and 95% similarity.

### Analysis of fragment size by gel electrophoresis

A PCR amplification of the region containing the central direct repeat (DRIII) (Mendez-Rios et al., 2012) was carried out with the primers forward (5′-CCCGTTCCGTTGATAGAT-3′) and reverse (5′-GGAAGAAGTACAATCTCTA-3′). The PCR product was run in a 2% agarose gel with a 100 bp ladder (Invitrogen) as marker. The most intense band of each isolate was considered for the estimation of the number of repeats. The sources of ECTV isolates used as templates have been previously described (Smith and Alcami, 2000).

### Genome analysis and annotation

The genomes were annotated using Artemis (Wellcome Trust Sanger Institute) (Rutherford et al., 2000). Percentages of identity and number of different nucleotides (nt) between the genomes were obtained by full-length genome alignment using Geneious Pro 6.1 (Biomatters). Open reading frames (ORFs) that code for non-overlapping proteins with a minimum 120 nt, with similarity to a poxvirus ortholog of known function or with preserved domains were annotated as genes. Fragmented genes, generated by deletion or insertion, with similarity to poxvirus functional orthologs were annotated as pseudogenes. Genes and pseudogenes were named with a three letter acronym EVN or EVC (the first two letters standing for ECTV and the third letter indicating the initial of the Naval or Cornell isolate) and numbered consecutively from left to right region of the genome. Pseudogenes were indicated with a P after the number. The schematic representation of the ECTV-Naval genome was designed with the Artemis DNAPlotter and refined manually.

### Experimental infection of susceptible BALB/c and DBA/2 mice

Groups of five female BALB/c and DBA/2 mice, six to seven weeks old, were purchased from Charles River Laboratories, anesthetized using isoflurane and infected subcutaneously in the left hind footpad with 1, 10 or 10^2^ pfu of semi-purified ECTV-Naval, ECTV-Cornell, ECTV-Moscow or PBS as control. Clinical signs of illness and body weight loss were monitored daily for 19 days post-infection (dpi). Mice were housed under biological safety level 3 in a ventilated rack (Allentown) with free access to food and water and 12 h light/12 h dark cycle. Animal experiments were approved by the Ethical Committee of the Consejo Superior de Investigaciones Científicas and the Biological Safety Department of the Centro de Biología Molecular Severo Ochoa, and carried out according to European regulations.

## Nucleotide sequence accession number

The genome sequence of ECTV-Naval was submitted to GenBank with the accession number KJ563295.

## Footnotes

Appendix A Supplementary data associated with this article can be found in the online version at doi:10.1016/j.virol.2014.06.010.

## References

[bib1] Afonso P.P., Silva P.M., Schnellrath L.C., Jesus D.M., Hu J., Yang Y., Renne R., Attias M., Condit R.C., Moussatché N., Damaso C.R. (2012). Biological characterization and next-generation genome sequencing of the unclassified Cotia virus SPAn232 (Poxviridae). J. Virol..

[bib2] Alcami A., Symons J.A., Collins P.D., Williams T.J., Smith G.L. (1998). Blockade of chemokine activity by a soluble chemokine binding protein from vaccinia virus. J. Immunol..

[bib3] Alejo A., Pontejo S.M., Alcami A. (2011). Poxviral TNFRs: properties and role in viral pathogenesis. Adv. Exp. Med. Biol..

[bib4] Allen A.M., Clarke G.L., Ganaway J.R., Lock A., Werner R.M. (1981). Pathology and diagnosis of mousepox. Lab. Anim. Sci..

[bib5] Almazan F., Tscharke D.C., Smith G.L. (2001). The vaccinia virus superoxide dismutase-like protein (A45R) is a virion component that is nonessential for virus replication. J. Virol..

[bib6] Alzhanova D., Edwards D.M., Hammarlund E., Scholz I.G., Horst D., Wagner M.J., Upton C., Wiertz E.J., Slifka M.K., Früh K. (2009). Cowpox virus inhibits the transporter associated with antigen processing to evade T cell recognition. Cell Host Microbe.

[bib7] Andrewes C.H., Elford W.J. (1947). Infections ectromelia; experiments on interference and immunization. Br. J.Exp. Pathol..

[bib8] Baxby D. (1982). The surface antigens of orthopoxviruses detected by cross-neutralization tests on cross-absorbed antisera. J. Gen. Virol..

[bib9] Bonfield J.K., Smith K., Staden R. (1995). A new DNA sequence assembly program. Nucl. Acids Res..

[bib10] Bowie A., Kiss-Toth E., Symons J.A., Smith G.L., Dower S.K., O’Neill L.A. (2000). A46R and A52R from vaccinia virus are antagonists of host IL-1 and toll-like receptor signaling. Proc. Natl. Acad. Sci. USA.

[bib11] Buller R. (2004). Mousepox: a small animal model for biodefense research. Appl. Biosaf..

[bib12] Byun M., Wang X., Pak M., Hansen T.H., Yokoyama W.M. (2007). Cowpox virus exploits the endoplasmic reticulum retention pathway to inhibit MHC class I transport to the cell surface. Cell Host Microbe.

[bib13] Cao J.X., Teoh M.L., Moon M., McFadden G., Evans D.H. (2002). Leporipoxvirus Cu-Zn superoxide dismutase homologs inhibit cellular superoxide dismutase, but are not essential for virus replication or virulence. Virology.

[bib14] Chen N., Danila M.I., Feng Z., Buller R.M.L., Wang C., Han X., Lefkowitz E.J., Upton C. (2003). The genomic sequence of ectromelia virus, the causative agent of mousepox. Virology.

[bib15] Dick E.J., Kittell C.L., Meyer H., Farrar P.L., Ropp S.L., Esposito J.J., Buller R.M., Neubauer H., Kang Y.H., McKee A.E. (1996). Mousepox outbreak in a laboratory mouse colony. Lab. Anim. Sci..

[bib16] Dixon L.W. (1981). Control of mousepox epizootics in St Louis and Chicago. Lab. Anim. Sci..

[bib17] Esteban D.J., Buller R.M. (2004). Identification of residues in an orthopoxvirus interleukin-18 binding protein involved in ligand binding and species specificity. Virology.

[bib18] Esteban D.J., Buller R.M.L. (2005). Ectromelia virus: the causative agent of mousepox. J. Gen. Virol..

[bib19] Favier A.-L., Flusin O., Lepreux S., Fleury H., Labrèze C., Georges A., Crance J.-M., Boralevi F. (2011). Necrotic ulcerated lesion in a young boy caused by cowpox virus infection. Case Rep. Dermatol..

[bib20] Fenner F. (1981). Mousepox (infectious ectromelia): past, present, and future. Lab. Anim. Sci..

[bib21] Fenner F., Wittek R., Dumbell K.R. (1989). The Orthopoxviruses..

[bib22] Giulio D.B.D., Eckburg P.B. (2004). Rev. Human Monkeypox.

[bib23] Graham K.A., Lalani A.S., Macen J.L., Ness T.L., Barry M., Liu L.Y., Lucas A., Clark-Lewis I., Moyer R.W., McFadden G. (1997). The T1/35 kDa family of poxvirus-secreted proteins bind chemokines and modulate leukocyte influx into virus-infected tissues. Virology.

[bib24] Groppel K. (1962). The occurrence of ectromelia (mousepox) in wild mice. Arch. Exp. Vet..

[bib25] Harte M.T., Haga I.R., Maloney G., Gray P., Reading P.C., Bartlett N.W., Smith G.L., Bowie A., O׳Neill L.A. (2003). The poxvirus protein A52R targets Toll-like receptor signaling complexes to suppress host defense. J. Exp. Med..

[bib26] Huggins J., Goff A., Hensley L., Mucker E., Shamblin J., Wlazlowski C., Johnson W., Chapman J., Larsen T., Twenhafel N., Karem K., Damon I.K., Byrd C.M., Bolken T.C., Jordan R., Hruby D (2009). Nonhuman primates are protected from smallpox virus or monkeypox virus challenges by the antiviral drug ST-246. Antimicrob.Agents Chemother..

[bib27] Ichihashi Y., Matsumoto S. (1966). Studies on the nature of Marchal bodies (A-type inclusion) during ectromelia virus infection. Virology.

[bib28] La Regina M.C., Doyle R.E. (1981). Mousepox at St Louis University--preliminary report. Lab. Anim. Sci..

[bib1001] Lefkowitz E.J., Wang C., Upton C. (2006). Poxviruses: past, present and future. Virus Res..

[bib29] Lin Z., Wang X., Strong M.J., Concha M., Baddoo M., Xu G., Baribault C., Fewell C., Hulme W., Hedges D., Taylor C.M., Flemington E.K. (2013). Whole-genome sequencing of the Akata and Mutu Epstein-Barr virus strains. J. Virol..

[bib30] Lipman N.S., Nguyen H., Perkins S. (1999). Mousepox: a threat to mouse colonies. Lab. Anim..

[bib31] Lipman N.S., Perkins S., Nguyen H., Pfeffer M., Meyer H. (2000). Mousepox resulting from use of ectromelia virus-contaminated, imported mouse serum. Comp. Med..

[bib32] Loparev V.N., Parsons J.M., Knight J.C., Panus J.F., Ray C.A., Buller R.M., Pickup D.J., Esposito J.J. (1998). A third distinct tumor necrosis factor receptor of orthopoxviruses. Proc. Natl. Acad. Sci. USA.

[bib33] Lopez-Bueno A., Tamames J., Velazquez D., Moya A., Quesada A., Alcami A. (2009). High diversity of the viral community from an Antarctic lake. Science.

[bib34] Mahnel H. (1983). [Disinfection for viruses]. Zentralblatt für Veterinärmedizin. Reihe B. J. Vet. Med. Series B.

[bib35] Marchal J. (1930). Infectious ectromelia. A hitherto undescribed virus disease of mice. J.Pathology Bacteriol..

[bib36] Mavian C., Lopez-Bueno A., Alcami A. (2014). Genome Sequence of WAU86/88-1, a new variant of vaccinia virus lister strain from Poland. Genome Announc.

[bib37] Mavian C., Lopez-Bueno A., Balseiro A., Casais R., Alcami A., Alejo A. (2012). The genome sequence of the emerging common midwife toad virus identifies an evolutionary intermediate within ranaviruses. J. Virol..

[bib38] Mavian C., López-Bueno A., Fernández Somalo M.P., Alcamí A., Alejo A. (2012). Complete genome sequence of the European sheatfish virus. J. Virol..

[bib39] Mendez-Rios J.D., Martens C.a., Bruno D.P., Porcella S.F., Zheng Z.-M., Moss B. (2012). Genome sequence of erythromelalgia-related poxvirus identifies it as an ectromelia virus strain. PloS One.

[bib40] Osterrieder N., Meyer H., Pfeffer M. (1994). Characterization of the gene encoding the A-type inclusion body protein of mousepox virus. Virus Genes.

[bib41] Qin L., Liang M., Evans D.H. (2013). Genomic analysis of vaccinia virus strain TianTan provides new insights into the evolution and evolutionary relationships between Orthopoxviruses. Virology.

[bib42] Radford A.D., Chapman D., Dixon L., Chantrey J., Darby A.C., Hall N. (2012). Application of next-generation sequencing technologies in virology. J. Gen. Virol..

[bib43] Reyes A., Haynes M., Hanson N., Angly F.E., Heath A.C., Rohwer F., Gordon J.I. (2010). Viruses in the faecal microbiota of monozygotic twins and their mothers. Nature.

[bib44] Rubio D., Xu R.H., Remakus S., Krouse T.E., Truckenmiller M.E., Thapa R.J., Balachandran S., Alcami A., Norbury C.C., Sigal L.J. (2013). Crosstalk between the type 1 interferon and nuclear factor kappa B pathways confers resistance to a lethal virus infection. Cell Host Microbe.

[bib45] Rutherford K., Parkhill J., Crook J., Horsnell T., Rice P., Rajandream M.A., Barrell B. (2000). Artemis: sequence visualization and annotation. Bioinformatics.

[bib46] Sakala I.G., Chaudhri G., Buller R.M., Nuara A.a., Bai H., Chen N., Karupiah G. (2007). Poxvirus-encoded gamma interferon binding protein dampens the host immune response to infection. J. Virol..

[bib47] Satheshkumar P.S., Moss B. (2009). Characterization of a newly identified 35-amino-acid component of the vaccinia virus entry/fusion complex conserved in all chordopoxviruses. J. Virol..

[bib48] Seet B.T., Johnston J.B., Brunetti C.R., Barrett J.W., Everett H., Cameron C., Sypula J., Nazarian S.H., Lucas A., McFadden G. (2003). Poxviruses and immune evasion. Annu. Rev. Immunol..

[bib49] Shchelkunov S.N. (2012). Orthopoxvirus genes that mediate disease virulence and host tropism. Adv. Virol..

[bib50] Smith C.A., Smith T.D., Smolak P.J., Friend D., Hagen H., Gerhart M., Park L., Pickup D.J., Torrance D., Mohler K., Schooley K., Goodwin R.G. (1997). Poxvirus genomes encode a secreted, soluble protein that preferentially inhibits beta chemokine activity yet lacks sequence homology to known chemokine receptors. Virology.

[bib51] Smith G.L., McFadden G. (2002). Smallpox: anything to declare? Nature reviews. Immunology.

[bib52] Smith V.P., Alcami A. (2000). Expression of secreted cytokine and chemokine inhibitors by ectromelia virus. J. Virol..

[bib53] Smith V.P., Alcami A. (2002). Inhibition of interferons by ectromelia virus. J. Virol..

[bib54] Streisinger G., Okada Y., Emrich J., Newton J., Tsugita A., Terzaghi E., Inouye M. (1966). Frameshift mutations and the genetic code. Cold Spring Harb. Symp. Quant. Biol..

[bib55] Wallace G.D. (1981). Mouse pox threat. Science.

[bib56] Wolfs T.F., Wagenaar J.A., Niesters H.G., Osterhaus A.D. (2002). Rat-to-human transmission of Cowpox infection. Emerg. Infect. Dis..

[bib57] Xiang Y., Moss B. (1999). IL-18 binding and inhibition of interferon gamma induction by human poxvirus-encoded proteins. Proc. Natl. Acad. Sci. USA.

[bib58] Xu R.H., Cohen M., Tang Y., Lazear E., Whitbeck J.C., Eisenberg R.J., Cohen G.H., Sigal L.J. (2008). The orthopoxvirus type I IFN binding protein is essential for virulence and an effective target for vaccination. J. Exp. Med..

[bib59] Yu E., Zhai D., Jin C., Gerlic M., Reed J.C., Liddington R. (2011). Structural determinants of caspase-9 inhibition by the vaccinia virus protein, F1L. J. Biol. Chem..

[bib60] Zheng Z.M., Specter S., Zhang J.H., Friedman H., Zhu W.P. (1992). Further characterization of the biological and pathogenic properties of erythromelalgia-related poxviruses. J Gen. Virol..

[bib61] Zheng Z.M., Zhang J.H., Hu J.M., Liu S.F., Zhu W.P. (1988). Poxviruses isolated from epidemic erythromelalgia in China. Lancet.

