# Supplementary material for: The genome sequence of ectromelia virus Naval and Cornell isolates from outbreaks in North America
Source: Virology. 2014 Aug;462-463:218–26. doi: 10.1016/j.virol.2014.06.010 (PMC4139192; doi:10.1016/j.virol.2014.06.010)
Supplement: Supplementary file 1 — Supplementary data [file mmc1.doc]

Table S1. Predicted ECTV Naval genes and pseudogenes

| **ECTV-Naval genes and pseudogenes** | | | | | **OPV orthologs** | | | |
| --- | --- | --- | --- | --- | --- | --- | --- | --- |
| **ORF** | **Position (bp)** | **bp** | **aa** | **Function/Characteristics** | **ECTV-Moscow** | **VACV-Cop** | **CPXV-BR** | **VARV-BSH** |
| EVN001 | 2,035-2,775 c | 741 | 247 | 35k, chemokine binding protein, secreted | EVM001 | C23L | CPXV003 | G3R |
| EVN002P | 2,911-3,021 c |  |  | CrmB/TNF and chemokine binding protein, SECRET domain |  | B28R | CPXV005 | G2R |
| EVN003 | 3,108-4,868 c | 1,761 | 587 | Ankyrin like protein, NF-*k*B inhibitor | EVM002 | C21L | CPXV006 | G1R |
| EVN004P | 5,075-5,324 c |  |  | Ankyrin like protein | A | C9L | CPXV008 | D9L |
| EVN005P | 5,321-5,503 c |  |  | CrmE/secreted TNF binding protein | B |  |  |  |
| EVN006 | 5,580-6,539 c | 960 | 320 | CrmD, TNF receptor and SECRET domain (chemokine binding), secreted | EVM003 |  | CPXV221 |  |
| EVN007P | 6,549-7,381 c |  |  | Ankyrin like protein | C | B18R | CPXV211 | B16R |
| EVN008 | 7,518-8,339 c | 822 | 273 | Kelch like protein | EVM004 |  |  |  |
| EVN009 | 8,458-10,410 c | 1,953 | 650 | Modulation of the ubiquitination machinery, ankyrin like protein | EVM005 |  | CPXV011 |  |
| EVN010P | 10,442-10,934 c |  |  | Lectin like protein | EVM006/EVM007 |  | CPXV012 |  |
| EVN011P | 11,013-11,183 c |  |  |  |  |  | CPXV013 |  |
| EVN012 | 11,256-11,864 c | 609 | 202 | SECRET domain, secreted | EVM008 |  | CPXV005 |  |
| EVN013 | 11,861-12,196 c | 336 | 111 | CD30 homolog, secreted | EVM009 |  | CPXV015 |  |
| EVN014 | 12,287-14,578 c | 2,292 | 763 | Ankyrin like protein | EVM010 | B18R | CPXV016 | B16R |
| EVN015P | 15,050-15,274 c |  |  | Ankyrin like protein | E | C9L | CPXV017 | D9L |
| EVN016 | 15,451-15,699 | 249 | 83 | Viral growth factor, secreted | EVM010,5 | C11R | CPXV021 | D4R |
| EVN017 | 16,016-17,011 c | 996 | 331 |  | EVM011 | C10L | CPXV022 | D5L |
| EVN018 | 17,522-18,250 | 729 | 242 | E3, ubiquitin ligase, zinc finger | EVM012 |  | CPXV023 | D6R |
| EVN019 | 18,401-18,781 c | 381 | 126 | IL-18 binding protein, secreted | EVM013 |  | CPXV024 | D7L |
| EVN020P | 18,843-20,332 c |  |  | Ankyrin like protein, CHOhr | F | C9L | CPXV025 | D9L |
| EVN021 | 20,471-20,686 c | 216 | 72 |  | EVM014 |  | CPXV026 |  |
| EVN022P | 20,934-21,619 c |  |  |  | G | C9L | CPXV027 | D9L |
| EVN023P | 21,621-22,060 c |  |  |  | H | C8L | CPXV028 |  |
| EVN024 | 22,126-22,578 c | 453 | 150 | Host range, ankyrin like protein | EVM015 | C7L | CPXV029 | D11L |
| EVN025 | 22,806-23,273 c | 468 | 155 |  | EVM016 | C6L | CPXV030 | D12L |
| EVN026P | 23,712-24,029 c |  |  |  | I | C5L | CPXV032 | D13L |
| EVN027P | 24,091-24,621 c |  |  |  | J | C4L | CPXV033 | D14L |
| EVN028 | 24,659-25,447 c | 789 | 262 | Complement binding protein, secreted | EVM017 | C3L | CPXV034 | D15L |
| EVN029 | 25,503-27,041 c | 1,539 | 512 | Kelch like protein, virulence factor | EVM018 | C2L | CPXV035 | D16L |
| EVN030P | 27,138-27,723 c |  |  |  | K | C1L | CPXV036 | D18L |
| EVN031 | 27,728-28,081 c | 354 | 117 | Apoptotic inhibitor and NF-*k*B activation factor | EVM019 | N1L | CPXV037 | P1L |
| EVN032 | 28,210-28,743 c | 534 | 177 | Alpha-amanitin sensitivity | EVM020 | N2L | CPXV038 | N2L |
| EVN033 | 28,776-30,194 c | 1,419 | 472 | Ankyrin like protein | EVM021 | M1L | CPXV039 | O1L |
| EVN034P | 30,175-30,846 c |  |  |  | L | M2L | CPXV040 | O2L |
| EVN035 | 30,981-31,838 c | 858 | 285 | Host range, ankyrin like protein | EVM022 | K1L | CPXV041 | O3L/C1L |
| EVN036 | 32,063-33,184 c | 1,122 | 373 | SPI-3, serin inhibitor protease | EVM023 | K2L | CPXV042 | K3L |
| EVN037P | 33,251-33,474 c |  |  | eIF-2α homolog | M | K3 | CPXV043 | C3L |
| EVN038 | 33,525-34,799 c | 1,275 | 424 | DNAase, nicking and joining function, phospholipase D like protein | N | K4L | CPXV044 |  |
| EVN039 | 34,821-35,651 c | 831 | 176 | Lysophospholipase like protein | EVM024 | K5L | CPXV045 |  |
| EVN040P | 35,821-36,224 |  |  | Phospholipase D | O | K7 | CPXV046 | C4L |
| EVN041 | 36,300-37,385 c | 1,278 | 425 | Caspase 9 inhibitor, Bcl-2 family | EVM025 | F1L | CPXV048 | C5L |
| EVN042 | 37,385-37,828 c | 444 | 147 | Deoxyuridine triphosphatase | EVM026 | F2L | CPXV049 | C6L |
| EVN043 | 37,864-39,309 c | 1,446 | 481 | Kelch like protein, virulence factor | EVM027 | F3L | CPXV050 | C7L |
| EVN044 | 39,320-40,279 c | 960 | 319 | Ribonucleotide reductase, small subunit | EVM028 | F4L | CPXV051 | C8L |
| EVN045P | 40,319-41,274 c |  |  |  | P | F5L | CPXV052 | C9L |
| EVN046 | 41,312-41,527 c | 216 | 72 |  | EVM029 | F6L | CPXV053 | C10L |
| EVN047 | 41,545-41,784 c | 240 | 80 |  | EVM030 | F7L | CPXV054 | C11L |
| EVN048 | 41,935-42,129 c | 195 | 65 |  | EVM031 | F8L | CPXV055 | C12L |
| EVN049 | 42,187-42,825 c | 639 | 212 | Membrane protein | EVM032 | F9L | CPXV056 | C13L |
| EVN050 | 42,812-44,131 c | 1,320 | 439 | Serine threonine kinase | EVM033 | F10L | CPXV057 | C14L |
| EVN051 | 44,154-45,218 c | 1,065 | 354 |  | EVM034 | F11L | CPXV059 | C15L |
| EVN052 | 45,277-47,181 c | 1,905 | 634 | EEV maturation | EVM035 | F12L | CPXV060 | C16L |
| EVN053 | 47,214-48,332 c | 1,119 | 372 | P37, major envelope protein (EEV), phospholipase D like protein | EVM036 | F13L | CPXV061 | C17L |
| EVN054 | 48,353-48,565 c | 213 | 71 |  | EVM037 | F14L | CPXV062 | C18L |
| EVN055 | 48,830-49,306 c | 477 | 158 |  | EVM038 | F15L | CPXV064 | C19L |
| EVN056 | 49,313-50,008 c | 696 | 231 |  | EVM039 | F16L | CPXV065 | C20L |
| EVN057 | 50,070-50,375 | 306 | 101 | VP11, structural protein (IMV) | EVM040 | F17R | CPXV066 | C21R |
| EVN058 | 50,372-51,811 c | 1,440 | 479 | Poly A polymerase, large subunit | EVM041 | E1L | CPXV067 | E1L |
| EVN059 | 51,808-54,021 c | 2,214 | 737 |  | EVM042 | E2L | CPXV068 | E2L |
| EVN060 | 54,127-54,699 c | 573 | 190 | IFN resistance factor | EVM043 | E3L | CPXV069 | E3L |
| EVN061 | 54,755-55,534 c | 780 | 259 | RNA polymerase, 30kDa subunit | EVM044 | E4L | CPXV070 | E4L |
| EVN062 | 55,583-56,608 | 1,026 | 341 |  | EVM045 | E5R | CPXV071 | E5R |
| EVN063 | 56,805-58,508 | 1,704 | 567 | Morphogenesis factor | EVM046 | E6R | CPXV072 | E6R |
| EVN064 | 58,589-59,086 | 498 | 165 |  | EVM047 | E7R | CPXV073 |  |
| EVN065 | 59,214-60,035 | 822 | 273 | Membrane protein, transcription and morphogenesis factor | EVM048 | E8R | CPXV074 | E8R |
| EVN066 | 60,042-63,062 c | 3,021 | 1,006 | DNA polymerase | EVM049 | E9L | CPXV075 | E9L |
| EVN067 | 63,094-63,378 | 285 | 95 | Membrane protein, morphogenesis factor, disulphide bridge formation | EVM050 | E10R | CPXV076 | E10R |
| EVN068 | 63,376-63,765 c | 390 | 129 |  | EVM051 | E11L | CPXV077 | E11L |
| EVN069 | 63,752-65,752 c | 2,001 | 666 | Membrane protein | EVM052 | O1L | CPXV078 | Q1L |
| EVN070 | 65,796-66,122 c | 327 | 108 | Glutaredoxin homolog | EVM053 | O2L | CPXV079 | Q2L |
| EVN071 | 66,149-66,253 c | 105 | 35 | Cell entry |  | O3L |  |  |
| EVN072 | 66,266-67,204 c | 939 | 312 | DNA binding, late phase viral morphogenesis | EVM054 | I1L | CPXV080 | K1L |
| EVN073 | 67,214-67,432 c | 219 | 73 |  | EVM055 | I2L | CPXV081 | K2L |
| EVN074 | 67,433-68,242 c | 810 | 269 | dsDNA binding phosphoprotein | EVM056 | I3L | CPXV082 | K3L |
| EVN075 | 68,325-70,640 c | 2316 | 771 | Ribonucleotide reductase, large subunit | EVM057 | I4L | CPXV083 | K4L |
| EVN076 | 70,670-70,906 c | 237 | 79 | Membrane protein, structural protein (IMV) | EVM058 | I5L | CPXV084 | K5L |
| EVN077 | 70,925-72,073 c | 1,149 | 382 |  | EVM059 | I6L | CPXV085 | K6L |
| EVN078 | 72,066-73,337 c | 1,272 | 423 | Cysteine protease, morphogenesis factor | EVM060 | I7L | CPXV086 | K7L |
| EVN079 | 73,343-75,373 | 2,031 | 676 | Nucleoside triphosphate phosphohydrolase II, RNA/DNA helicase | EVM061 | I8R | CPXV087 | K8R |
| EVN080 | 75,376-77,151 c | 1,776 | 591 | Viral envelope protein (IMV), metalloprotease | EVM062 | G1L | CPXV088 | H1L |
| EVN081 | 77,148-77,483 c | 336 | 111 | Cell entry protein | EVM063 | G3L | CPXV089 | H3L |
| EVN082 | 77,477-78,139 | 663 | 220 | VLTF, viral late transcription factor | EVM064 | G2R | CPXV090 | H2R |
| EVN083 | 78,109-78,483 c | 375 | 124 | Glutaredoxin 2 homolog | EVM065 | G4L | CPXV091 | H4L |
| EVN084 | 78,486-79,790 | 1,305 | 434 | Morphogenesis factor | EVM066 | G5R | CPXV092 | H5R |
| EVN085 | 79,798-79,986 | 189 | 63 | RNA polymerase, 7kDa subunit | EVM067 | G5,5R | CPXV093 | H5,5R |
| EVN086 | 79,991-80,488 | 498 | 165 |  | EVM068 | G6R | CPXV094 | H6R |
| EVN087 | 80,453-81,568 c | 1,116 | 371 | Structural protein (IMV) | EVM069 | G7L | CPXV095 | H7L |
| EVN088 | 81,599-82,381 | 783 | 260 | VLTF-1, viral late transcription factor | EVM070 | G8R | CPXV096 | H8R |
| EVN089 | 82,401-83,423 | 1,023 | 340 | Cell entry myristoylated protein | EVM071 | G9R | CPXV097 | H9R |
| EVN090 | 83,424-84,176 | 753 | 250 | Structural protein 25 kDa (IMV) | EVM072 | L1R | CPXV098 | M1R |
| EVN091 | 84,208-84,468 | 261 | 87 | Morphogenesis factor | EVM073 | L2R | CPXV099 | M2R |
| EVN092 | 84,461-85,501 c | 1,041 | 346 | Early activation factor | EVM074 | L3L | CPXV100 | M3L |
| EVN093 | 85,526-86,281 | 756 | 251 | DNA/RNA binding protein | EVM075 | L4R | CPXV101 | M4R |
| EVN094 | 86,291-86,677 | 387 | 128 | Cell entry protein | EVM076 | L5R | CPXV102 | M5R |
| EVN095 | 86,634-87,095 | 462 | 153 | Structural protein (IMV) | EVM077 | J1R | CPXV103 | L1R |
| EVN096 | 87,111-87,644 | 534 | 177 | Thymidine kinase | EVM078 | J2R | CPXV104 | L2R |
| EVN097 | 87,709-88,710 | 1,002 | 333 | Vp39, poly A polymerase, small multifunctional subunit | EVM079 | J3R | CPXV105 | L3R |
| EVN098 | 88,625-89,182 | 558 | 185 | Rpo22, RNA polymerase, 22 kDa subunit | EVM080 | J4R | CPXV106 | L4R |
| EVN099 | 89,247-89,648 c | 402 | 133 | Membrane protein | EVM081 | J5L | CPXV107 | L5L |
| EVN100 | 89,754-93,614 | 3,861 | 1,286 | Rpo147, RNA polymerase, 147 kDa subunit | EVM082 | J6R | CPXV108 | L6R |
| EVN101 | 93,611-94,126 c | 516 | 171 | Serine/tyrosine phosphatase, IFN signalling evasion | EVM083 | H1L | CPXV109 | I1L |
| EVN102 | 94,140-94,709 | 570 | 189 | Cell entry protein | EVM084 | H2R | CPXV111 | I2R |
| EVN103 | 94,712-95,686 c | 975 | 324 | Immunodominant envelope protein (IMV) | EVM085 | H3L | CPXV112 | I3L |
| EVN104 | 95,687-98,071 c | 2,385 | 794 | RAP94, RNA-polymerase associated protein | EVM086 | H4L | CPXV113 | I4L |
| EVN105 | 98,257-98,895 | 639 | 212 | VLTF-4, viral late transcription factor | EVM087 | H5R | CPXV114 | I5R |
| EVN106 | 98,896-99,840 | 945 | 314 | DNA topoisomerase I | EVM088 | H6R | CPXV115 | I6R |
| EVN107 | 99,877-100,317 | 441 | 146 | Membrane protein | EVM089 | H7R | CPXV117 | I7R |
| EVN108 | 100,359-102,890 | 2,532 | 843 | Capping enzyme, large subunit | EVM090 | D1R | CPXV118 | F1R |
| EVN109 | 102,849-103,289 | 441 | 146 | Core structural protein (IMV) | EVM091 | D2L | CPXV119 | F3L |
| EVN110 | 103,282-103,995 | 714 | 237 | Core structural protein (IMV) | EVM092 | D3R | CPXV120 | F2R |
| EVN111 | 103,995-104,651 | 657 | 218 | DNA Glycosylase | EVM093 | D4R | CPXV121 | F4R |
| EVN112 | 104,683-107,040 | 2,358 | 785 | Nucleoside-triphosphatase | EVM094 | D5R | CPXV122 | F5R |
| EVN113 | 107,081-108,994 | 1,914 | 637 | Viral early transcription factor | EVM095 | D6R | CPXV123 | F6R |
| EVN114 | 109,021-109,506 | 486 | 161 | Rpo18, RNA-polymerase, 18 kDa subunit | EVM096 | D7R | CPXV124 | F7R |
| EVN115 | 109,469-110,383 c | 915 | 304 | Envelope structural protein (IMV), cell entry protein | EVM097 | D8L | CPXV125 | F8L |
| EVN116 | 110,425-111,066 | 642 | 213 | 25 kDa mut-T-like protein, mRNA level regulation | EVM098 | D9R | CPXV126 | F9R |
| EVN117 | 111,063-111,815 | 753 | 250 | 29 kDa kDa mut-T-like protein, mRNA level regulation | EVM099 | D10R | CPXV127 | F10R |
| EVN118 | 111,812-113,707 c | 1,896 | 631 | NPHI, DNA-dependent ATPase | EVM100 | D11L | CPXV128 | N1L |
| EVN119 | 113,741-114,604 c | 864 | 287 | VITF, viral initiation transcription factor, capping | EVM101 | D12L | CPXV129 | N2L |
| EVN120 | 114,635-116,290 c | 1,656 | 551 | Membrane structural protein, resistance to rifampin (IMV) | EVM102 | D13L | CPXV131 | N3L |
| EVN121 | 116,314-116,766 c | 453 | 150 | VLTF-2, viral late transcription factor | EVM103 | A1L | CPXV132 | A1L |
| EVN122 | 116,787-117,461 c | 675 | 224 | VLTF-3, viral late transcription factor | EVM104 | A2L | CPXV133 | A2L |
| EVN123 | 117,461-117,688 c | 228 | 76 | Disulphide bridge formation pathway protein | EVM105 | A2,5L | CPXV134 |  |
| EVN124 | 117,703-119,637 c | 1,935 | 644 | P4b, mayor core protein (IMV) | EVM106 | A3L | CPXV135 | A4L |
| EVN125 | 119,690-120,535 c | 846 | 281 | Immunodominant structural core protein (IMV) | EVM107 | A4L | CPXV136 | A5L |
| EVN126 | 120,573-121,067 | 495 | 164 | Rpo19, RNA-polymerase 19 kDa subunit | EVM108 | A5R | CPXV137 | A6R |
| EVN127 | 121,064-122,182 | 1,119 | 372 | Morphogenesis factor | EVM109 | A6L | CPXV138 | A7L |
| EVN128 | 122,206-124,338 c | 2,133 | 710 | VETF, viral early transcription factor, 82 kDa large subunit | EVM110 | A7L | CPXV139 | A8L |
| EVN129 | 124,392-125,258 | 867 | 288 | VITF-3, viral intermediate transcription factor | EVM111 | A8R | CPXV140 | A9R |
| EVN130 | 125,255-125,581 c | 327 | 108 | Morphogenesis factor (IMV) | EVM112 | A9L | CPXV141 | A10L |
| EVN131 | 125,582-128,257 c | 2,676 | 891 | 4a, major core protein (IMV) | EVM113 | A10L | CPXV142 | A11L |
| EVN132 | 128,272-129,228 | 957 | 318 | Morphogenesis factor | EVM114 | A11R | CPXV143 | A12R |
| EVN133 | 129,230-129,805 c | 576 | 191 | Morphogenesis factor, core protein (IMV) | EVM115 | A12L | CPXV144 | A14L |
| EVN134 | 129,832-130,029 c | 198 | 66 | P8, structural membrane core protein (IMV) | EVM116 | A13L | CPXV145 | A14L |
| EVN135 | 130,141-130,410 c | 270 | 90 | P16, structural membrane protein (IMV) | EVM117 | A14L | CPXV146 | A15L |
| EVN136 | 130,430-130,588 c | 159 | 53 | Structural membrane protein (IMV) | EVM117,5 | A14,5L | CPXV147 |  |
| EVN137 | 130,581-130,862 c | 282 | 94 | Morphogenesis factor | EVM118 | A15L | CPXV148 | A16L |
| EVN138 | 130,846-131,979 c | 1,134 | 377 | 35 kDa myristoylated protein, cell entry | EVM119 | A16L | CPXV149 | A17L |
| EVN139 | 131,982-132,590 c | 609 | 202 | Structural membrane protein (IMV), morphogenesis factor | EVM120 | A17L | CPXV150 | A18L |
| EVN140 | 132,605-134,086 | 1,482 | 493 | DNA helicase, DNA dependent ATPase | EVM121 | A18R | CPXV151 | A19R |
| EVN141 | 134,070-134,300 c | 231 | 77 |  | EVM122 | A19L | CPXV152 | A20L |
| EVN142 | 134,301-134,657 c | 357 | 118 | Cell entry protein | EVM123 | A21L | CPXV153 | A22L |
| EVN143 | 134,656-135,936 | 1,281 | 426 | DNA-polymerase processivity factor | EVM124 | A20R | CPXV154 | A21R |
| EVN144 | 135,866-136,429 | 564 | 187 | Holliday junction resolvase | EVM125 | A22R | CPXV155 | A23R |
| EVN145 | 136,449-137,597 | 1,149 | 382 | VITF-3, viral intermediate transcription factor | EVM126 | A23R | CPXV156 | A24R |
| EVN146 | 137,594-141,088 | 3,495 | 1,164 | Rpo132, RNA-polymerase, 132 kDa subunit | EVM127 | A24R | CPXV157 | A25R |
| EVN147 | 141,081-144,422 c | 3,342 | 1,113 | ATIp, A-type inclusion bodies protein (ATIs) | EVM128 | A25L | CPXV158 | A26L |
| EVN148P | 144,468-145,979 c |  |  | P4c/ ATIs inclusion factor | Q | A26L | CPXV159 | A30L |
| EVN149 | 146,031-146,363 c | 333 | 110 | A-type inclusion bodies protein, bridge protein between ATIp and P4c | EVM129 | A27L | CPXV162 | A31L |
| EVN150 | 146,364-146,804 c | 441 | 146 | Cell entry protein | EVM130 | A28L | CPXV163 | A31,5L |
| EVN151 | 146,805-147,722 c | 918 | 305 | Rpo35, RNA-polymerase, 35 kDa subunit | EVM131 | A29L | CPXV164 | A32L |
| EVN152 | 147,688-147,918 c | 231 | 77 | Morphogenesis factor | EVM132 | A30L | CPXV165 | A33L |
| EVN153 | 148,078-148,458 | 381 | 126 |  | EVM133 | A31R | CPXV166 | A24R |
| EVN154 | 148,428-149,237 c | 810 | 269 | ATPase | EVM134 | A32L | CPXV167 | A25L |
| EVN155 | 149,355-149,912 | 558 | 185 | Structural envelope glycoprotein (EEV) | EVM135 | A33R | CPXV168 | A26R |
| EVN156 | 149,936-150,442 | 507 | 168 | Structural envelope glycoprotein (EEV), similar to CD69, virulence factor | EVM136 | A34R | CPXV169 | A37R |
| EVN157 | 150,485-151,015 | 531 | 176 | MHC-II expression inhibitor | EVM137 | A35R | CPXV171 |  |
| EVN158 | 151,083-151,565 | 483 | 160 | Morphogenesis factor, virion protein (EEV) | EVM137,5 | A36R | CPXV172 | A38R |
| EVN159P | 151,807-152,569 |  |  |  | R | A37R | CPXV173 | A40R |
| EVN160 | 152,848-153,681 c | 834 | 277 | Protein similar to CD47, extracellular Ca^2+^ entry | EVM138 | A38L | CPXV173 | A41L |
| EVN161 | 153,696-154,895 | 1,200 | 399 | Viral semaphorin, secreted | EVM139 | A39R | CPXV175 | A42R/A43R |
| EVN162P | 154,920-155,405 |  |  | Lectin like protein | S | A40R | CPXV177 |  |
| EVN163 | 155,517-156,188 c | 672 | 223 | Chemokine binding protein, secreted | EVM140 | A41L | CPXV176 | A44L |
| EVN164 | 156,350-156,754 | 405 | 134 | Profilin, intracellular viral protein transport | EVM141 | A42R | CPXV178 | A45R |
| EVN165 | 156,794-157,429 | 636 | 211 |  | EVM142 | A43R | CPXV179 | A46R |
| EVN166 | 157,731-158,771 c | 1,041 | 346 | 3β-hydroxysteroid dehydrogenase | EVM143 | A44L | CPXV180 | A47L |
| EVN167P | 158,819-159,118 |  |  | SOD | EVM144 | A45R | CPXV183 |  |
| EVN168 | 159,185-159,907 | 723 | 240 | NF-*k*B inhibitor, similar to the TLR/IL-1R cytoplasmic domain | EVM145 | A46R | CPXV184 | A49R |
| EVN169 | 159,994-160,728 | 735 | 244 |  | EVM146 | A47L | CPXV185 | J1L |
| EVN170 | 160,827-161,441 | 615 | 204 | Motif A of the ATP/GTP binding site | EVM147 | A48R | CPXV186 | J2R |
| EVN171P | 161,491-161,841 |  |  |  | T | A49R | CPXV187 | J3R |
| EVN172 | 162,014-163,678 | 1,665 | 554 | DNA ligase | EVM148 | A50R | CPXV188 | J4R |
| EVN173 | 163,731-164,735 | 1,005 | 334 |  | EVM149 | A51R | CPXV189 | J5R |
| EVN174P | 164,806-165,189 |  |  | NF-*k*B inhibitor | U | A52R | CPXV190 |  |
| EVN175P | 165,710-166,231 |  |  | CrmC/secreted TNF binding protein | V | A53R | CPXV191 |  |
| EVN176 | 166,506-168,197 | 1,692 | 563 | Modulation of the ubiquitination machinery, kelch like protein | EVM150 | A55R | CPXV193 | J6R |
| EVN177 | 168,247-169,092 | 846 | 281 | Viral hemoagglutinin | EVM151 | A56R | CPXV194 | J7R |
| EVN178P | 169,110-169,699 |  |  | Guanylate kinase | W | A57R | CPXV195 | J8R |
| EVN179 | 169,860-170,759 | 900 | 299 | 30 kDa serin-threonine kinase | EVM152 | B1R | CPXV196 | B1R |
| EVN180 | 170,826-172,337 | 1,512 | 503 | Schlafen homolog | EVM153 |  | CPXV197 |  |
| EVN181 | 172,574-174,268 | 1,695 | 564 | Modulation of the ubiquitination machinery, ankyrin like protein | EVM154 | B4R | CPXV198 | B5R |
| EVN182 | 174,355-175,308 | 954 | 317 | Structural envelope glycoprotein (EEV) | EVM155 | B5R | CPXV199 | B6R |
| EVN183 | 175,467-176,000 | 534 | 177 |  | EVM156 | B6R | CPXV200 | B7R |
| EVN184 | 176,039-176,584 | 546 | 181 | SECRET domain secreted | EVM157 | B7R | CPXV201 |  |
| EVN185 | 176,639-177,439 | 801 | 266 | IFN-γ receptor, secreted | EVM158 | B8R | CPXV202 | B8R |
| EVN186P | 177,551-178,350 |  |  | Kelch like protein | X | B10R | CPXV204 |  |
| EVN187 | 178,471-178,734 | 264 | 87 | Serin-threonine kinase | EVM159 | B11R | CPXV205 |  |
| EVN188 | 178,800-179,660 | 861 | 286 | Serin-threonine kinase | EVM160 | B12R | CPXV206 | B11R |
| EVN189 | 179,752-180,786 | 1,035 | 344 | SPI-2, serine protease inhibitor | EVM161 | B14R | CPXV207 | B12R |
| EVN190 | 180,865-181,359 | 495 | 164 |  | EVM162 | B15R | CPXV208 | B13R |
| EVN191 | 181,446-182,432 | 987 | 328 | IL-1-β receptor, secreted | EVM163 | B16R | CPXV209 |  |
| EVN192 | 182,487-183,509 c | 1,023 | 340 |  | EVM164 | B17L | CPXV210 | B17L |
| EVN193 | 183,599-185,383 | 1,785 | 594 | Ankyrin like protein | EVM165 | B18R | CPXV211 | B16R |
| EVN194 | 185,444-186,520 | 1,077 | 358 | IFN-a/β binding protein, secreted | EVM166 | B19R | CPXV212 | B17R |
| EVN195P | 186,617-188,997 |  |  | Ankyrin like protein | Y | B20R | CPXV213 | B18R |
| EVN196 | 189,113-190,792 | 1,680 | 559 | P65, modulation of the ubiquitination machinery, kelch like protein | EVM167 | A55R | CPXV215 | J6R |
| EVN197 | 191,051-192,163 | 1,113 | 370 | SPI-1, serine protease inhibitor | EVM168 | C12L | CPXV217 | B21R |
| EVN198P | 192,337-192,891 |  |  |  | Z | C14L | CPXV218 |  |
| EVN199 | 193,154-198,928 | 5,775 | 1,924 | Membrane protein | EVM169 |  | CPXV219 | B22R |
| EVN200P | 199,206-200,883 |  |  | Ankyrin like protein | CA | B18R | CPXV220 | B16R |
| EVN201 | 200,890-201,852 | 963 | 320 | CrmD, TNF receptor and SECRET domain, secreted | EVM170 |  | CPXV221 |  |
| EVN202P | 201,926-202,108 |  |  | CrmE/secreted TNF binding protein | BA |  |  |  |
| EVN203P | 202,105-202,354 |  |  | Ankyrin like protein | AA | C9L | CPXV223 | D9L |
| EVN204 | 202,561-204,324 | 1,764 | 587 | Ankyrin like protein, NF-*k*B inhibitor | EVM171 | B27R | CPXV225 | G1R |
| EVN205P | 204,408-204,518 |  |  | CrmB/TNF and chemokine binding protein, SECRET domain |  | B28R | CPXV226 | G2R |
| EVN206 | 204,654-205,397 | 744 | 247 | 35k, chemokine binding protein, secreted | EVM172 | B29R |  | G3R |

aa, number of amino acids; VACV-COP: vaccinia Copenhagen (M35027.1), CPXV-BR: cowpox Brighton Red (AF482758.2), VARV-BSH: Variola Bangladesh (L22579.1); BTB, (Broad-Complex, Tramtrack and Bric a brac) also known as the POZ domain (Poxvirus and zinc finger); IMV, intracellular mature virion; IEV, intracellular enveloped virion; EEV, extracellular enveloped virion; PKR, dsRNA-dependent protein kinase; VLTF, viral late transcription factor; VITF, viral intermediate transcription factor; IEV, intracellular enveloped virion;
