# Supplementary material for: The genome sequence of ectromelia virus Naval and Cornell isolates from outbreaks in North America
Source: Virology. 2014 Aug;462-463:218–26. doi: 10.1016/j.virol.2014.06.010 (PMC4139192; doi:10.1016/j.virol.2014.06.010)
Supplement: Supplementary file 2 — Supplementary data [file mmc2.doc]

Table S2. Sequence differences between ECTV-Moscow and ECTV-Naval

| **ECTV-Moscow** | **ECTV-Naval** | **Amino acid differences*** |
| --- | --- | --- |
| EVM001 | EVN001 | L170I |
| EVM002 | EVN003 | V21A, A223V, S446F |
| EVM003 | EVN006 | F263L |
| EVM004 | EVN008 | G102D, E184Q |
| EVM005 | EVN009 | P26S, N32S, L120R, K177Q, N160D, T182S, G205S, G244S, C272R |
| EVM006/ EVM007 | EVN010P | **Pseudogene** |
| EVM008 | EVN012 | I93V, V164A |
| EVM009 | EVN013 | M70T |
| EVM010 | EVN014 | I57R, K556N |
| EVM010,5 | EVN016 | A10T |
| EVM011 | EVN017 | D5V |
| EVM012 | EVN018 | T146/ |
| EVM013 | EVN019 | T88I, E122N, Y123I, L124W, A125L, E126K, Del (127-138) |
| EVM014 | EVN021 | Del (1-10), Ins (41-44) de GVNG |
| EVM016 | EVN025 | D86N |
| EVM017 | EVN028 | D229Y, K255E |
| EVM022 | EVN035 | S32T |
| EVM023 | EVN036 | G292S |
| Region N | EVN038 | Ad (187-424) **Gene** |
| EVM024 | EVN039 | V85I, Del (253) de H |
| EVM025 | EVN041 | V14I, Ins (456) de G |
| EVM027 | EVN042 | Y395H, G407D, I480M, R481E |
| EVM028 | EVN044 | I258V |
| EVM029 | EVN046 | R22K, F71L, T72Q |
| EVM030 | EVN047 | N21, K22 |
| EVM031 | EVN048 | D9G |
| EVM034 | EVN051 | N235K |
| EVM042 | EVN059 | A69S, E379D |
| EVM047 | EVN064 | K76E |
| EVM048 | EVN065 | T3A |
| EVM049 | EVN066 | S738P |
| EVM059 | EVN077 | I64V |
| EVM060 | EVN078 | S82G |
| EVM061 | EVN079 | S116L, M387I |
| EVM062 | EVN080 | I398M |
| EVM063 | EVN081 | A66V |
| EVM064 | EVN082 | D144G |
| EVM066 | EVN084 | R352H |
| EVM068 | EVN086 | N144D |
| EVM069 | EVN087 | T228A |
| EVM074 | EVN092 | L263I, I343T |
| EVM075 | EVN093 | I181T |
| EVM112 | EVN130 | Ins (94-95) de NS |
| EVM113 | EVN131 | G236R, A881V |
| Region Q | EVN148P | **Pseudogene** |
| EVM144 | EVN167P | N96M, D97I, Q98R, R99M, Del (100-125) **Pseudogene** |
| EVM145 | EVN168 | P67S |
| EVM150 | EVN176 | D358N |
| EVM151 | EVN177 | D139Y |
| EVM154 | EVN181 | M389V |
| EVM166 | EVN194 | D144E |
| EVM167 | EVN196 | V241M |

Del, Deletion; Ins, Insertion; Ad, Addition.
